# Role of Inflammation in Classification of Diabetic Macular Edema by Optical Coherence Tomography

**DOI:** 10.1155/2019/8164250

**Published:** 2019-12-20

**Authors:** Yoo-Ri Chung, Young Ho Kim, Seong Jung Ha, Hye-Eun Byeon, Chung-Hyun Cho, Jeong Hun Kim, Kihwang Lee

**Affiliations:** ^1^Department of Ophthalmology, Ajou University School of Medicine, Suwon, Republic of Korea; ^2^Institute of Medical Science, Ajou University School of Medicine, Suwon, Republic of Korea; ^3^Vascular Microenvironment Laboratory, Department of Pharmacology, Seoul National University College of Medicine, Seoul, Republic of Korea; ^4^Ischemic/Hypoxic Disease Institute, Seoul National University College of Medicine, Seoul, Republic of Korea; ^5^Cancer Research Institute, Seoul National University College of Medicine, Seoul, Republic of Korea; ^6^Fight against Angiogenesis-Related Blindness (FARB) Laboratory, Clinical Research Institute, Seoul National University Hospital, Seoul, Republic of Korea; ^7^Department of Biomedical Sciences, Seoul National University College of Medicine, Seoul, Republic of Korea; ^8^Department of Ophthalmology, Seoul National University College of Medicine, Seoul, Republic of Korea

## Abstract

Diabetic macular edema (DME) is the abnormal accumulation of fluid in the subretinal or intraretinal spaces in the macula in patients with diabetic retinopathy and leads to severely impaired central vision. Technical developments in retinal imaging systems have led to many advances in the study of DME. In particular, optical coherence tomography (OCT) can provide longitudinal and microstructural analysis of the macula. A comprehensive review was provided regarding the role of inflammation using OCT-based classification of DME and current and ongoing therapeutic approaches. In this review, we first describe the pathogenesis of DME, then discuss the classification of DME based on OCT findings and the association of different types of DME with inflammation, and finally describe current and ongoing therapeutic approaches using OCT-based classification of DME. Inflammation has an important role in the pathogenesis of DME, but its role appears to differ among the DME phenotypes, as determined by OCT. It is important to determine how the different DME subtypes respond to intravitreal injections of steroids, antivascular endothelial growth factor agents, and other drugs to improve prognosis and responsiveness to treatment.

## 1. Introduction

Diabetic retinopathy (DR) is a major microvascular complication of diabetes and a leading cause of visual impairment in the working-age population [1–4]. The prevalence of DR among patients with diabetes is greater than 40%, and approximately 5-10% of these individuals have vision-threatening conditions [3, 4]. Hyperglycemia activates cytokines and growth factors and leads to dysfunction of vascular and neuronal cells. This increases oxidative stress and inflammation, stimulates the protein kinase C and polyol pathways, and increases the production of advanced glycation end products [5, 6]. The inflammatory response itself enhances these same pathways, resulting in leukostasis and increased cell permeability due to the increased production of vascular endothelial growth factor (VEGF) [7]. Several studies reported significantly increased systemic and local expression of proinflammatory cytokines in the retinas of patients with DR [7–9]. These proinflammatory molecules contribute to structural and functional abnormalities of the retina and adversely affect endothelial cells, pericytes, Müller cells, and microglial cells [10].

Diabetic macular edema (DME) is characterized by the abnormal accumulation of fluid in the subretinal or intraretinal spaces of the macula and leads to severely impaired central vision [11]. Technical developments in retinal imaging have greatly improved the study of DME. For example, optical coherence tomography (OCT) provides longitudinal and microstructural analysis of the macula [11]. Several factors contribute to the pathogenesis of DME, including hypoxia and oxidative stress, upregulation of VEGF, alteration of the blood-retinal barrier (BRB), retinal vessel leukostasis, pericyte loss, and vascular hyperpermeability [12, 13]. In this review, we focus on the pathogenic effect of inflammation in DME as determined by OCT. We first review the pathogenesis of DME and then discuss the use of OCT for classification of DME and current and ongoing therapeutic approaches based on OCT classification.

## 2. Pathogenesis of DME

### 2.1. The Healthy Blood-Retinal Barrier

The healthy retina is an immune-privileged organ because of the BRB, which consists of inner and outer layers. Under normal physiological conditions, the BRB regulates fluid entry and drainage of molecules and maintains the retina in a dehydrated and transparent state [11]. Breakdown of the BRB occurs during early DR and leads to localized vascular hyperpermeability and retinal edema [10, 13].

**Figure 1:**
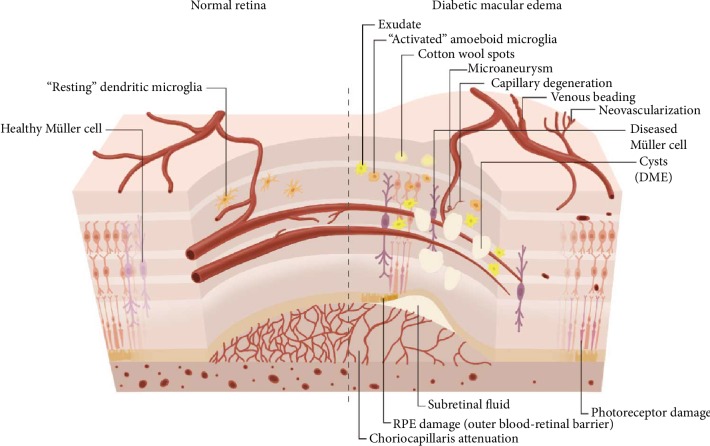
Schematic illustration of the BRB of a healthy retina compared with DME. DME exhibits vascular changes including microaneurysms, capillary degeneration, venous beading, neovascularization, associated with activated microglia, Müller cell swelling, retinal pigment epithelium RPE damage, and choriocapillaris attenuation. Breakdown of BRB results in subretinal fluid and retinal cysts.

The inner BRB has tight junctions (zonula occludens) between the endothelial cells of retinal vessels, allowing interactions with pericytes and smooth muscle cells [14]. Transmembrane proteins, scaffolding proteins, and signaling proteins form endothelial tight junctions [11]. Adherens junctions connect the cytoskeleton of pericytes to endothelial cells, allowing molecular signaling between these different cells [11]. A pericyte-derived lipidic mediator modulates the inner BRB [15], so pericytes have a critical role in the maintenance of the BRB. The processes of glial cells also wrap around retinal capillaries [11]. Retinal Müller cells and astrocytes ensheath vascular plexuses and stabilize the tight junctions between endothelial cells [16]. Microglia contribute to the maintenance of the inner BRB by producing soluble factors that are important for vesicular communication (Figure 1) [13, 17].

The intercellular junction complex of the retinal pigment epithelium (RPE) forms the outer layer of the BRB [18]. In particular, the basolateral membrane of the RPE faces Bruch's membrane and separates the neurosensory retina from the fenestrated endothelium of the choriocapillaris [18, 19]. The RPE junction complex is formed by tight, adherens, and gap junctions, which control the transport of fluids and solutes and maintain the integrity of the retina [18, 19].

### 2.2. Glial Dysfunction in DR

Activation of Müller cells and microglia initiates retinal inflammation [20, 21]. Microglia constantly monitor the physiological microenvironment, and their activation is a hallmark of the inflammatory process during acute injury [22]. Microglia can detect early signs of hyperglycemia and seem to participate in all stages of DR after activation [20, 23].

Retinal Müller cells are specialized macroglial cells that regulate the tightness of the BRB by controlling the intercellular transport of ions, water, and bicarbonate [24]. Thus, they function as a structural backbone for the retina, regulate vascular homeostasis, and provide metabolic support for neural activity [24]. The role of Müller cells in controlling movement of water and ions allows them to buffer intraretinal increases of potassium ions [25]. Ischemia and inflammation can alter the potassium channels of Müller cells and cause them to accumulate intracellular fluid [24, 26]. Retinal Müller cells also produce VEGF, tumor necrosis factor alpha (TNF-*α*), interleukin- (IL-) 1*β*, and prostaglandins in the presence of inflammation and hypoxia, all of which aggravate vascular hyperpermeability [12, 27]. In DR, Müller cells trigger retina inflammation through stimulation of cluster of differentiation (CD) 40 and indirectly promote inflammation of microglia through release of adenosine triphosphate [28].

### 2.3. Immune Cell Function and Inflammation in DR

Hyperglycemia leads to upregulation of intercellular adhesion molecule 1 (ICAM-1), which mediates leukocyte adhesion to the vascular endothelium, resulting in vascular damage and capillary nonperfusion [29]. Monocyte chemotactic protein 1 (MCP-1), a major chemotactic factor for monocytes, is also increased in the vitreous of patients with DR [29, 30]. The levels of several inflammatory markers, such as VEGF, sICAM-1, and MCP-1, are higher in the vitreous of patients with DME [30]. Noma et al. [30] reported that aqueous flare (an index of inflammation) correlated with the levels of inflammatory cytokines in the vitreous and suggested that inflammation increased vascular permeability and thereby disrupted the blood-aqueous barrier.

Leukocytes contribute to microvascular damage by releasing cytokines and superoxide and by physically blocking the capillaries [10]. They interact with ICAM-1 and vascular cell adhesion molecule on the surface of endothelial cells, resulting in adherence of blood cells to the endothelial wall (leukostasis) [10]. These molecular changes during inflammation contribute to neurodegeneration of the retina, as indicated by the migration of subunits of nuclear factor kappa B, a proinflammatory transcription factor, into the nuclei of retinal neurons in the presence of diabetes [10, 31].

### 2.4. Retinal Pigment Epithelial Cells in DR

The RPE forms the outer layer of the BRB and regulates the movement of solutes between the capillaries of the choroid and the photoreceptor layers [18]. RPE cells also play an important role in immune processes, in that they express major histocompatibility complex molecules, adhesion molecules, and cytokines [19]. DME is associated with RPE dysfunction and impaired transport of water from the subretinal space into the choriocapillaris [18, 19]. The healthy RPE also regulates the high oxidative stress of the retina, and DR impairs this function by reducing the levels of antioxidants [19].

## 3. Use of OCT to Classify and Characterize DME

### 3.1. Classification by OCT

**Figure 2:**
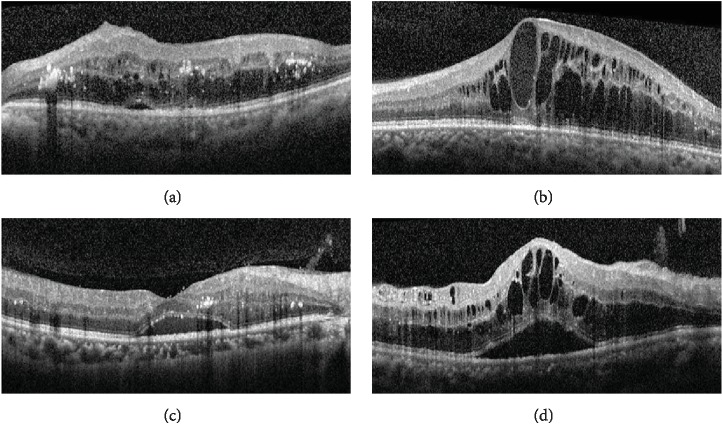
Representative OCT images of the different types of DME: (a) diffuse retinal thickening, (b) cystoid macular edema, (c) serous retinal detachment, and (d) mixed type.

Technical advances have increased the resolution of OCT, led to more precise quantification of the thickness of the retina and choroid, and provided more detailed information about the individual layers. Clinicians now commonly use OCT to classify the different types of DME: diffuse thickening type (sponge-like diffuse retinal thickening), cystoid macular edema (CME) type (thickening of fovea with intraretinal cystoid change), and serous retinal detachment (SRD) type (thickening of fovea with subretinal fluid) (Figure 2) [32, 33]. In DME, leakage from the deep capillary plexus induces fluid migration into the outer plexiform layer; leakage from the superficial capillary plexus leads to fluid accumulation in the inner nuclear layer [34]. The accumulation of subretinal fluid suggests alteration of the outer BRB, probably due to fluid movement from the retina through weakened and permeable external limiting membrane or from the increased permeability of the choriocapillaris through the dysfunctional RPE [33].

The diffuse thickening type of DME results from breakdown of the inner BRB due to inflammation and oxidative stress, which leads to increased vascular permeability [32, 35]. The intraretinal cysts present in the CME type of DME presumably originate from the liquefaction and necrosis of Müller cells and the production of prostaglandins and inflammatory cytokines [25, 32, 36]. However, leukostasis theory has been suggested by the development of OCT angiography which allows isolation of capillary plexi and visualization of capillary flow [25]. Ischemia and inflammation both lead actions of integrins and adhesion molecules facilitating migration of leukocytes. This would plug effectively capillary-sized vessels, which appear as decreased deep vascular plexus flow around cystoid spaces in OCT angiography [25]. The presence of cystoid spaces in regions with decreased or absent deep plexus suggests that the fluid removal is potentially mediated by deep plexus, not by retinal Müller cells [25].

The subretinal fluid of the SRD type of DME might be due to the increased permeability of the choriocapillaris because of the dysfunctional RPE at an early stage of disease and then from the breakdown of the outer BRB through the permeable external limiting membrane [37, 38]. The SRD type is clinically significant because it is associated with poor prognosis for vision, probably because of disruption of the external limiting membrane [39]. Clinical studies investigating the association of DME phenotypes with prognosis following intravitreal injections also use this OCT-based classification.

### 3.2. Evidence of Inflammation in Different Types of DME

Numerous studies investigated the association of inflammatory cytokines with different DME phenotypes (as determined by OCT) in an effort to better determine prognosis and to select the most appropriate drug for intravitreal injections. In particular, the intraocular levels of potent proangiogenic cytokine (VEGF) and proinflammatory cytokine (IL-6) are both increased in DME, but they are modulated differently, so intravitreal injections of anti-VEGF and steroids might lead to different responses [39, 40].

Analysis of the intravitreal concentrations of cytokines in the eyes with the SRD type of DME indicated elevated intravitreal concentrations of VEGF, IL-6, and IL-8 [41]. An elevated level of IL-6 had a strong association with the presence of the SRD type of DME, suggesting that inflammation has an important role in the development of this phenotype [39]. Disruption of the external limiting membrane in the SRD type of DME is accompanied by cellular damage, and this attracts scavenger cells to the retina, which in turn produce IL-6 [42]. Another possibility might be due to migration towards and accumulation of the activated microglia in the outer segments of the retinal layer, which could produce excess amount of IL-6 and lead to the collection of fluid in the subretinal space. Greater choroidal thickness is also present in the SRD type of DME, which suggests increased choriocapillaris permeability caused by excessive production of VEGF from dysfunctional RPE [33].

### 3.3. Hyperreflectivity in OCT and Microglial Activation

**Figure 3:**
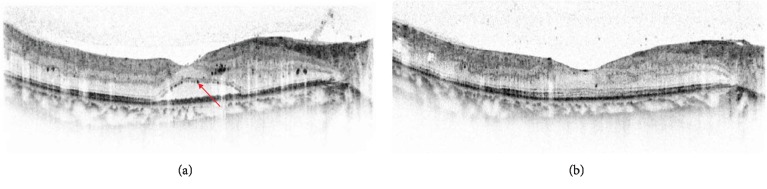
Representative OCT images (black-and-white mode) of hyperreflective foci (HF) in a patient with the SRD type of DME. (a) HF appear as intraretinal dark dots (*red arrow*). (b) Resolution of SRD and reduction of HF in the same patient after intravitreal injection of bevacizumab.

The increased image resolution provided by OCT allows detection of hyperreflective foci (HF), which appear as discrete intraretinal spots but differ from hard exudates (Figure 3) [43]. Some reports hypothesized that these HF were extravasated lipoproteins that precede the formation of hard exudates [38, 44], and one study hypothesized that HF represent microglial activation and migration [45]. Moreover, the number of HF is greater in patients with diabetes than in those without diabetes and greater in diabetic patients with DR than diabetic patients without DR [45]. This suggests that HF indicate the presence of microglial activation in the diabetic retina and are associated with the progression of DR [45]. A recent study investigating soluble CD14, a cytokine released by microglia and macrophage, reported that DME patients with diffuse edema showed higher levels of soluble CD14 in the aqueous humor as well as more HF in the inner retina than those with focal edema [46]. This suggests that HF observed on OCT might be due to activated microglia, associated with severe inflammatory reaction [46].

The SRD type of DME is associated with an increased number of HF and significant functional impairment of the macula [42]. The presence of HF is associated with poorer improvement of vision in patients with macular edema due to retinal vascular diseases, including DR [47, 48]. One study reported intraretinal HF in patients without detectable retinopathy, mainly in the inner retina initially, and this suggests an early alteration of microglia during pathogenesis of the diabetic retina [45]. The presence of more HF is also associated with poorer glycemic control in patients without significant DME, suggesting that HF could be useful as a marker for early-stage DR [49].

## 4. Therapy for Different Types of DME Based on OCT Classification

### 4.1. Conventional Treatment

Unfortunately, no available treatments target the early stages of DR (before the occurrence of sight-threatening complications), except for strict glycemic control [50, 51]. Intensive treatment of dyslipidemia with fenofibrate can reduce the rate of DR progression [50, 51], but there are controversies on the use of statin therapy, with some reports showing they had a protective effect [52, 53] and others reporting they increased the risk for development of diabetes [54–56].

Many clinicians have used focal/grid laser photocoagulation as a local treatment for DME to decrease the rate of severe vision loss, established in the Early Treatment Diabetic Retinopathy Study (ETDRS), the landmark randomized controlled trial [57, 58]. In a more recent clinical trial following ETDRS, focal/grid laser reduced moderate visual loss by 50% and led visual gain of ≥10 letters improvement in 28% of DME eyes receiving sham injection and prompt laser [59]. The exact therapeutic mechanism is unknown, but this procedure may destroy high-oxygen-consuming photoreceptors and restore function of the RPE [60]. However, the eyes with the diffuse thickening type of DME are less responsive to this treatment, and these patients only have limited improvement in vision [61]. In diffuse type of DME, the response to laser photocoagulation was limited, with 61% showing unchanged vision, 24% presenting visual deterioration, and only 15% showing visual improvement [61]. It is often insufficient to restore already impaired vision due to the long-term atrophic changes from laser burns, and the resulting scotomas have led many clinicians to prefer intravitreal injections [62]. Although the use of anti-VEGF agents is now the preferred and most effective treatment modality, laser photocoagulation remains an important treatment in real-world clinical practice due to its low cost [58].

### 4.2. Anti-VEGF Agents

Intravitreal injections of anti-VEGF agents are replacing laser photocoagulation as the standard treatment for most patients with DME [63]. All currently available anti-VEGF agents (ranibizumab, bevacizumab, and aflibercept) provide improvements in vision, while ranibizumab and aflibercept appear more effective for eyes with poorer baseline visual acuity [64–66]. These DME treatments also slow the progression of DR, and aflibercept provides more improvement in patients with proliferative DR at baseline [67].

In terms of OCT-based morphologic findings, bevacizumab appears effective in the diffuse thickening type of DME [68, 69], and one study reported that it was also effective for the CME type of DME [70]. In SRD type which is associated with external limiting membrane as well as RPE impairment, DME did not respond well to anti-VEGF agents [69, 71]. Ranibizumab is effective for the diffuse thickening type of DME, and fewer injections are needed [71]. Diffuse retinal thickening may originate from increased vascular permeability due to breakdown of the inner BRB, and this may explain the efficacy of these anti-VEGF agents.

### 4.3. Steroid Drugs

Various studies have reported the use of steroids as a treatment for DME. Corticosteroids may improve DME by targeting different pathways than those targeted by anti-VEGF agents [62]. Intravitreal injection of triamcinolone and a dexamethasone injectable implant are widely used corticosteroid treatments for macular edema from retinal vascular diseases, including DR [62]. Intravitreal triamcinolone injections were effective for the resolution of cysts in the CME type of DME [72]. The intraretinal cysts in this type of DME arise from liquefaction and necrosis of Müller cells, related to the production of prostaglandins and inflammatory cytokines, so the greater efficacy of steroids may be because they more effectively reduce the swelling of Müller cells [21, 25, 73].

In a prospective case series, intravitreal injection of triamcinolone seemed to be also more effective than anti-VEGF therapy in reducing macular thickness and improving vision in patients with the SRD type of DME [74]. This result needs cautious interpretation due to relatively short-term follow-up (24 weeks) [74], as long-term complications such as cataract and elevated intraocular pressure exist with intravitreal injection of triamcinolone [75, 76]. A recent study reported a good response to an injectable dexamethasone implant in the eyes with SRD, no HF, and a continuous ellipsoid zone at the fovea [48]. The SRD type of DME is also associated with hypoxia and inflammation, so this may explain the greater efficacy of steroids in this type of DME [74]. The SRD type of DME has higher concentrations of inflammatory cytokines in the aqueous humor and vitreous and also has increased levels of IL-6 [39]. Scavenger cells attracted by cellular damage to the external limiting membrane might be the source of IL-6 [42]. These findings may explain the efficacy of steroids in treatment of DME.

Because of the abundant evidence that inflammation has a major role in the pathogenesis of DME, there are many ongoing clinical trials using novel drugs that target inflammation (Table 1) [77].

## 5. Conclusions

Inflammation has an important role in the pathogenesis of DME, but its exact role appears to differ among the different DME phenotypes, as determined by OCT. It is important to determine how the different DME subtypes respond to intravitreal injections of steroids, anti-VEGF, and other drugs to improve patient prognosis and responsiveness to treatment. However, we are still far from a general consensus for OCT-based treatment regimens, so that further randomized controlled studies are needed to customize the best treatment for DME patients.

## Figures and Tables

**Table 1:** Agents currently in clinical trials as treatments for inflammation in diabetic macular edema.

Drug class	Molecule	Administration	Trial number	Trial status
Steroid	Betamethasone microsphere	Posterior sub-Tenon	NCT01411254	Phase 2/3 completed
	Dexamethasone-cyclodextrin	Topical	NCT01523314	Unknown
	Danazol	Oral	NCT02002403	Phase 2 completed
	NOVA63035	Intravitreal	NCT00665106	Phase 1 completed
TNF alpha inhibitor	Infliximab	Intravitreal	NCT00959725	Unknown
Tie-2 activator	AKB-9778	Subcutaneous	NCT02050828	Phase 2 completed
IGF-1 antagonist	Teprotumumab	Intravenous	NCT02103283	Phase 1 completed
Integrin antagonist	SF0166	Topical	NCT02914613	Phase 1/2 completed
mTOR inhibitor	Sirolimus	Subconjunctival	NCT00711490	Phase 1/2 completed

IGF = insulin-like growth factor; mTOR = mechanistic target of rapamycin; TNF = tumor necrosis factor.
